# Robust Antibody Responses to the BNT162b2 mRNA Vaccine Occur Within a Week After the First Dose in Previously Infected Individuals and After the Second Dose in Uninfected Individuals

**DOI:** 10.3389/fimmu.2021.722766

**Published:** 2021-08-26

**Authors:** Yosuke Hirotsu, Kenji Amemiya, Hiroki Sugiura, Miyuki Shinohara, Mika Takatori, Hitoshi Mochizuki, Masao Omata

**Affiliations:** ^1^Genome Analysis Center, Yamanashi Central Hospital, Kofu, Japan; ^2^Division of Genetics and Clinical Laboratory, Yamanashi Central Hospital, Kofu, Japan; ^3^Division of Clinical Biochemistry and Immunology, Yamanashi Central Hospital, Kofu, Japan; ^4^Division of Health Management, Yamanashi Central Hospital, Kofu, Japan; ^5^Division of Infection Control and Prevention, Yamanashi Central Hospital, Kofu, Japan; ^6^Central Clinical Laboratory, Yamanashi Central Hospital, Kofu, Japan; ^7^Department of Gastroenterology, Yamanashi Central Hospital, Kofu, Japan; ^8^The University of Tokyo, Tokyo, Japan

**Keywords:** SARS-CoV-2, COVID-19, antibody, mRNA vaccine, BNT162b2

## Abstract

**Background:**

Vaccines against severe acute respiratory syndrome coronavirus 2 can trigger acquired immunity in infection-naïve individuals and offer a path toward ending the coronavirus disease pandemic that began in 2019. However, the kinetics of early antibody responses in vaccinated individuals remain poorly understood.

**Method:**

We followed BNT162b2 mRNA-vaccinated health care workers (HCWs, N=108) including 103 infection-naïve and five previously infected individuals. A total of 763 blood samples were collected weekly or hourly basis before and after vaccination. Serological analysis of anti-spike and anti-nucleocapsid antibodies was performed.

**Results:**

Seroconversion occurred in all infection-naïve HCWs 3 weeks after the first dose (just before the second vaccination) and a marked boosting effect was observed at 4 weeks (1 week after the second dose). Among previously infected HCWs with pre-existing antibodies against the spike protein, a remarkable boosting effect was observed during the first week after vaccination, and a further increase in antibody titres was observed after the second dose. In one previously infected patient, daily blood sampling was conducted. Antibody titres began to increase 96 hours (4 days) after the first dose.

**Conclusion:**

The BNT162b2 mRNA vaccine remarkably enhanced antibody responses after the second dose in infection-naïve individuals and after the first dose in previously infected HCWs of all ages and genders. Antibody titres decreased slightly after the 5^th^ week post-vaccination. The robust boosting effect of immunisation suggests that increased antibody titres following exposure to the virus may restrict viral replication, prolong the incubation period, or lessen the severity of disease.

## Introduction

The rapid development of coronavirus disease 2019 (COVID-19) vaccines is now a global priority for public health. Widespread adaptive immunity against severe acute respiratory syndrome coronavirus 2 (SARS-CoV-2) is expected to contain the spread of the virus. Therefore, rapid implementation of vaccines is desirable. Vaccination programmes urgently need to be expanded, because of the number of new emergent lineages harbouring variants of concern. Although there is concern that the activity of antibodies elicited by vaccination or natural infection may be reduced by escape mutations in some lineages (1–7), mRNA vaccine-elicited antibodies are effective against these emerging lineages to some extent (8, 9).

The SARS-CoV-2 spike (S) glycoprotein forms a trimer that binds to angiotensin converting enzyme 2 and mediates cell entry (10). In particular, the receptor binding domain (RBD) of the S protein is highly immunogenic (11). COVID-19 mRNA vaccines, developed by Pfizer/BioNTech and Moderna, target the full-length S protein and induce an immune response through a two-dose prime-boost approach (12–14).

Phase 2/3 clinical trials of mRNA vaccines, including BNT162b2 (Pfizer/BioNTech) (12) and mRNA-1273 (Moderna) (13), have shown about 95% protection against SARS-CoV-2 infection and 100% efficacy in preventing severe COVID-19. The BNT162b2 vaccine reduced the incidence of symptomatic COVID-19, hospitalisation, severe illness, and mortality in a nationwide population study (15).

Previous reports showed that antibody titres were significantly increased after administration of COVID-19 mRNA vaccines. Anti-S antibody titres correlate with neutralising activity and increase with boosting (5, 16). Furthermore, the vaccine elicits humoral and cellular immune responses (17, 18). Vaccination elicits higher antibody responses in individuals previously infected with SARS-CoV-2 compared with infection-naïve individuals (19–23). A few studies have examined early antibody responses after vaccination (22, 24). However, there were no serological follow-up data from the same individuals, and variation in the early antibody response after vaccination by age and gender was not investigated. In this study, we aimed to characterise the kinetics of antibody titres in blood samples serially collected from infection-naïve and naturally infected healthcare workers (HCWs) after the first or second dose of BNT162b2 mRNA vaccine.

## Methods

### Participants and Samples

Written informed consent was obtained from all HCWs (N=108) who enrolled in this study. The cohort included 103 infection-naïve individuals and five HCWs who were previously infected with SARS-CoV-2. Among the overall study population, 35.2% (n=38) were women (mean age 46.3 years; range, 23–64 years) and 64.8% (n=70) were men (mean age 43.0 years; range, 23–75 years). Among the 103 infection-naïve HCWs (38 women and 65 men), 18 were in their 20s, 22 were in their 30s, 20 were in their 40s, 22 were in their 50s, and 21 were in their 60s or older. Among the five previously infected HCWs (all men), four were in their 20s and one was in his 30s.

Five HCWs were considered to have been previously infected in November 2020. SARS-CoV-2 test and serological test showed these individuals were PCR-positive, antigen-positive, and seropositive for anti-nucleocapsid (N) protein (25–29). These HCWs had been hospitalised during their prior infection; four had mild symptoms and one had moderate symptoms. The infection-naïve individuals had no history of PCR-positive tests for SARS-CoV-2 and were seronegative for anti-N protein antibody. The anti-N protein antibody titres of all HCWs were surveyed at different times (first survey in June 2020, second survey in December 2020, and 3–9 days before the first dose of vaccine).

All participants were inoculated with the BNT162b2 mRNA vaccine according to the standard prime-boost regimen. In brief, the BNT162b2 mRNA vaccine was administered *via* two doses (0.3 mL intramuscular injections of 30 µg) at 3-week intervals. The first dose was administered between 8 March and 12 March 2021 and the second dose was administered between 29 March and 2 April 2021. Peripheral blood samples were serially drawn from the 103 infection-naïve HCWs 3 to 9 days before the first dose of vaccine (baseline) and then 1, 3, 4, 5, and 7 weeks after the first dose. Blood samples were drawn from four of the five previously infected HCWs at baseline and then 1, 2, 3, 4, 5, and 7 weeks after the first dose. In one previously infected HCW, blood samples were drawn at baseline and then 6 and 12 hours as well as 1, 2, 3, 4, 5, 6, and 7 days after the first dose. At 3 weeks post-vaccination, blood was collected just before administration of the second dose.

### Serological Analysis

We measured the titre of anti-N protein antibody using the Elecsys Anti-SARS-CoV-2 antibody test (Roche Diagnostics, Basel, Switzerland) and the titre of anti-S protein RBD antibody using the Elecsys Anti-SARS-CoV-2 S antibody test (Roche Diagnostics) on a cobas^®^ 8000 automated platform (30). This assay utilises the electrochemiluminescence immunoassay principle. For anti-N antibody, samples with a <1.0 cut off index (COI) were considered negative, while those with a COI≥1.0 were considered positive. For anti-S antibody, samples containing <0.8 unit/mL (U/mL) were considered negative, while those containing ≥0.8 U/mL were considered positive following the manufacturer’s instructions.

### Ethics Statement

The Institutional Review Board of the Clinical Research and Genome Research Committee of Yamanashi Central Hospital approved this prospective study (Approval No. C2019-30 and C2020-14). Participation in the study was optional following informed consent. All study procedures were performed in accordance with the relevant guidelines and regulations and as set out in the Helsinki Declaration.

### Statistical Analysis

Statistical analyses (Kruskal-Wallis test, pairwise t-tests, and Student t-tests) were conducted using R version 3.6.2 (http://www.r-project.org/). Values of P < 0.05 were considered statistically significant.

## Results

### Kinetics of Antibody Titres in Infection-Naïve HCWs

**Figure 1:**
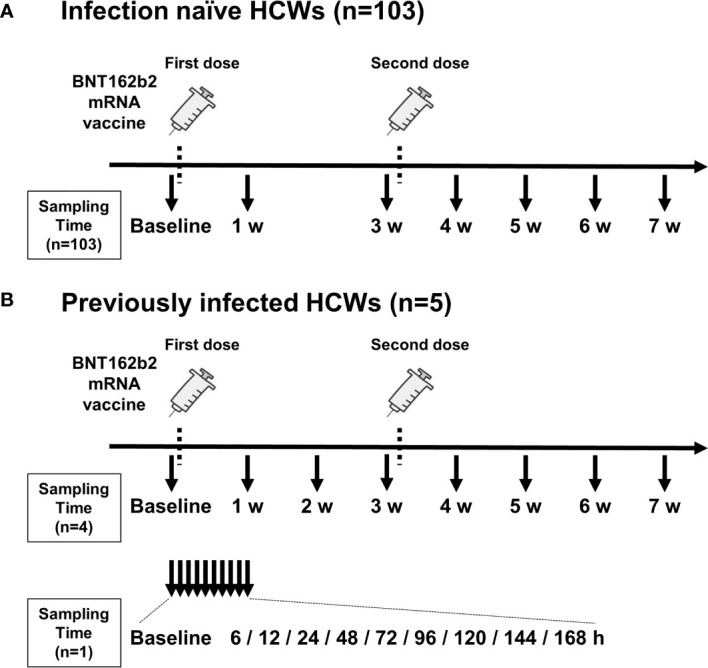
Timing of vaccination and blood sample collection. **(A)** Timing of blood sampling from 103 infection-naïve health care workers (HCWs). **(B)** Timing of blood sampling from five previously infected HCWs. Blood was sampled from four HCWs on a weekly basis (top) and from one HCW on an hourly basis (bottom). The timing of administration of two doses of BNT162b2 mRNA vaccine is indicated by dotted lines. Arrows indicate the timing of blood collection. Blood samples were collected before the first vaccination (baseline) and just before the second vaccination (3 w). w, week; h, hour.

**Figure 2:**
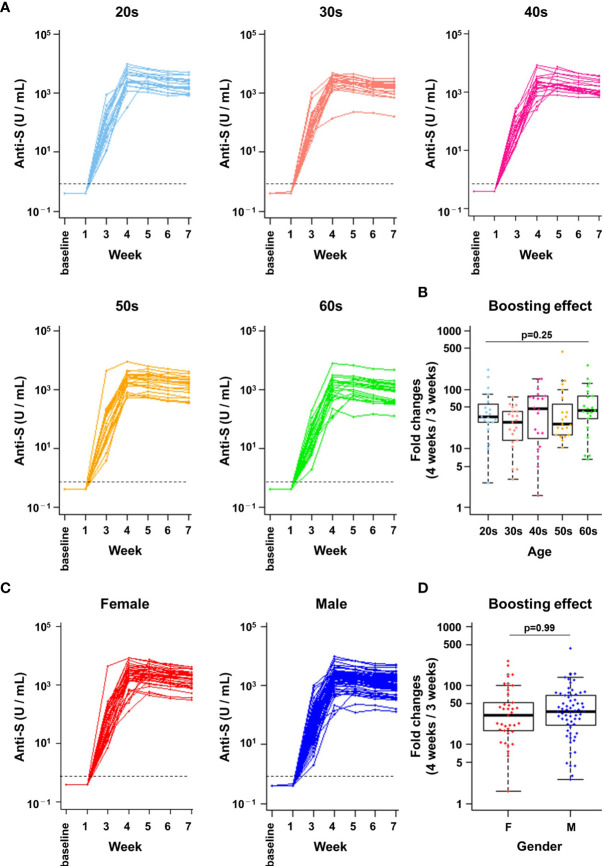
A robust boosting effect after the second vaccine dose was observed in individuals of all ages and genders. **(A, C)** Anti-spike (S) protein antibody titres in infection-naïve healthcare workers (n=103). The kinetics of antibody titres (U/mL) are indicated from baseline to 7 weeks after the first dose. Data by age group **(A)** and by gender **(C)** are shown. The dotted line indicates the cut-off value (0.8 U/mL). **(B, D)** Boosting effect before and after the second vaccine dose. The ratios of antibody titres immediately before (3 weeks) and 1 week after the second dose (4 weeks) are indicated. Data by age group **(B)** and by gender **(D)** are shown. There were no statistically significant differences in boosting effect between age groups (p = 0.25, Kruskal-Wallis test) or genders (p = 0.99, Student’s t-test). F, female; M, male.

To investigate the kinetics of antibody titres in infection-naïve HCWs after administration of the BNT162b2 mRNA vaccine, serial blood samples were drawn before vaccination (baseline) and at 1, 3, 4, 5, 6, and 7 weeks after the first dose (**Figure 1A**). All infection-naïve HCWs (n=103) were seronegative at baseline and 1 week after vaccination without any detectable increase in anti-S antibodies (**Figures 2A, C**). Although 100% (103/103) of infection-naïve HCWs showed seroconversion at 3 weeks after vaccination, the values of antibody titres were relatively low (median 57 U/mL, range: 2–991 U/mL) (**Figures 2A, C**). Titres of anti-S antibodies increased 138.3-fold on median (range: 4.9- to 2477.5-fold) at 3 weeks (one dose) compared to baseline (**Supplemental Figure 1**). There was no statistically significant difference in the ratio of antibody titre increase at 3 weeks after first dose compared to baseline for age and gender (age groups, p = 0.22, pairwise t-tests with Bonferroni adjustment; gender, p = 0.58, Student’s t-test, **Supplemental Figure 1**).

Notably, a marked boosting effect on the anti-S antibody titre was observed 1 week after the second dose (median 2177 U/mL, range: 108–9545 U/mL, **Figures 2A, C**). Titres of anti-S antibodies further increased 35.8-fold on median (range: 1.6- to 436.4-fold) at 4 weeks (two doses) compared to 3 weeks (one dose). No significant differences in boosting effect (ratio of 4 weeks *vs* 3 weeks) was observed between age groups (**Figure 2B**, p = 0.25, Kruskal-Wallis test). The median antibody increase was 54.6-fold for those in their 20s (range: 2.6- to 218.8-fold), 29.6-fold for those in their 30s (range: 3.0- to 73.9-fold), 53.9-fold for those in their 40s (range: 1.6- to 153.9-fold), 61.4-fold for those in their 50s (range: 10.5- to 436.4-fold), and 63.9-fold for those in their 60s or older (range: 6.5- to 262.1-fold). Furthermore, there were no significant differences in the anti-S antibody titre increase between females and males (31.2-fold and 37.0-fold, respectively) (**Figure 2D**, p = 0.99, Student’s t-test). Anti-S antibody titres peaked at 4 to 5 weeks in 98% of infection-naïve HCWs (4 weeks in 66% of HCWs [68/103] and 5 weeks in 32% of HCWs [33/103]) (**Figures 2A, C**). Thereafter, anti-S antibody titres started to decline at weeks 6 and 7. As expected, anti-N antibodies were undetectable over the study period in all infection-naïve HCWs (**Supplemental Figure 2**). These results showed that a remarkable boosting effect on anti-S antibody titres occurred after the second dose of the BNT162b2 mRNA vaccine regardless of age and gender.

### Differences in Early Antibody Responses by Age and Gender

**Figure 3:**
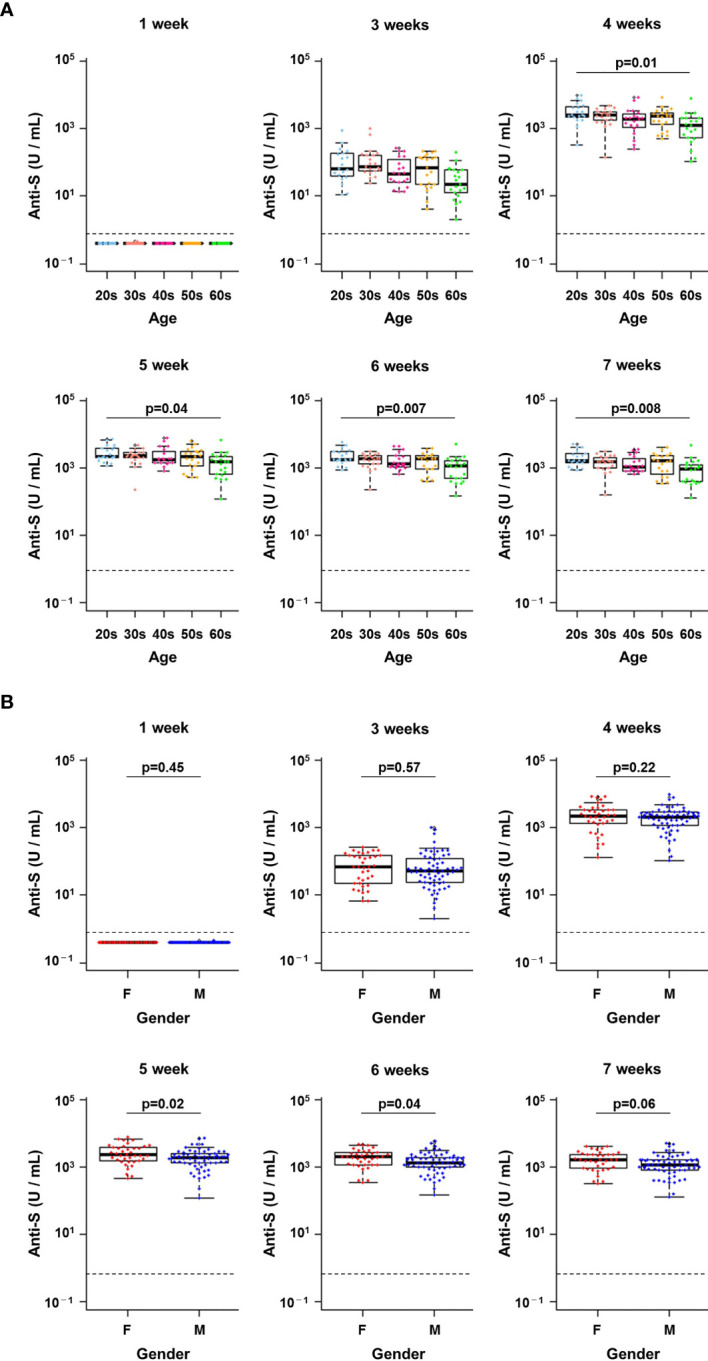
Changes in antibody titres at each week post-vaccination in infection-naïve healthcare workers. **(A, B)** Changes in the titres of anti-spike (S) antibodies from week 1 to week 7 after the first vaccine dose by age group **(A)** and by gender **(B)**. A pairwise t-test with Bonferroni correction was conducted for the multiple comparison test across the age groups. Student’s t-test was performed for statistical analyses between female (F) and male (M). The dotted line indicates the cut-off value (0.8 U/mL).

We next examined whether differences in the early antibody response to vaccination were associated with age or gender. There were no significant differences in anti-S protein antibody titres in HCWs of different age groups 1 week and 3 weeks after the first dose of BNT162b2 mRNA vaccine (**Figure 3A**). However, antibody titres at 4 weeks or later (i.e., after the second dose) were significantly higher for HCWs in their 20s compared with those in their 60s (p = 0.01 at 4 weeks, p = 0.04 at 5 weeks, p = 0.007 at 6 weeks and p = 0.008 at 6 weeks, pairwise t-tests with Bonferroni adjustment) (**Figure 3A**).

There were no significant differences in anti-S protein antibody titres between genders from 1 week to 4 weeks after the first dose (**Figure 3B**). In contrast, higher antibody titres were observed in women from weeks 5 to 7 (p = 0.02 at 5 weeks, p = 0.04 at 6 weeks, p = 0.06 at 6 weeks, Student’s t-tests) (**Figure 3B**). Thus, anti-S protein antibody titres after vaccination were slightly higher in younger HCWs and in women.

To further examine whether there were statistically significant differences by age and gender, stratified analysis was conducted. As mentioned above, in the overall analysis, there was a difference between those in their 20s and 60s (**Figure 3A**). Although we could not observe significant difference when further stratified by gender (**Supplemental Figures 3A, B**), it seems there is a trend of the age effect (i.e. the 20s were always higher than the 60s in female and male).

The overall analysis also showed that females tended to have higher antibody titres than males (**Figure 3B**), however, stratified analysis showed no significant difference in most of the comparison groups (**Supplemental Figures 3C–G**). These data indicated that while antibody titres induced by vaccination or infection may vary slightly by age and gender, the effects of boosting were similar among all HCWs.

### Kinetics of Antibody Responses in Previously Infected HCWs

**Figure 4:**
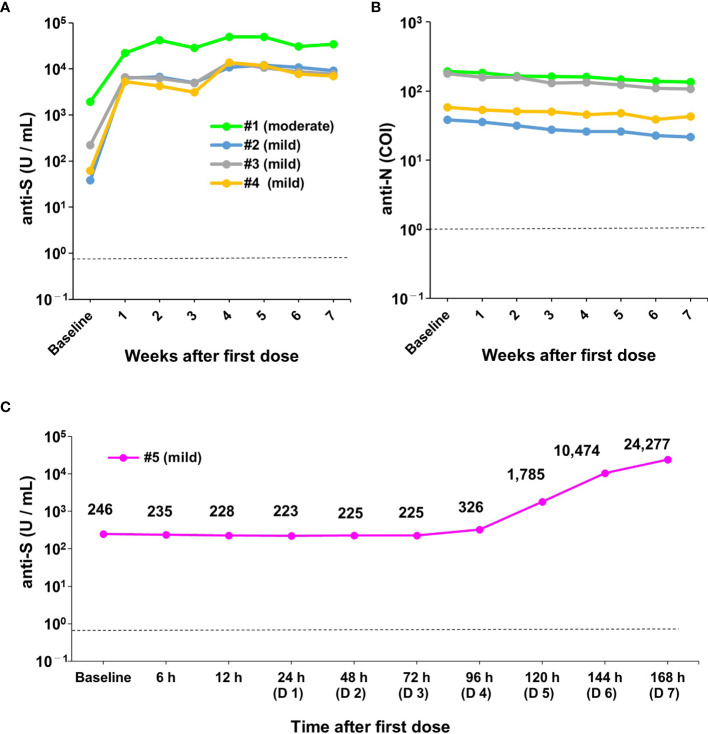
Robust humoral immune responses after the first dose of vaccine in previously infected healthcare workers. **(A, B)** Changes in antibody titres on a weekly basis (weeks 1 to 7) in previously infected healthcare workers (HCWs) (n=4) after the first dose of vaccine. Data show changes in anti-spike (S) **(A)** and anti-nucleocapsid (N) antibody titres **(B)**. During the previous infection, one of the four HCWs had moderate symptoms (individual #1) and three had mild symptoms (individuals #2, 3, and 4). **(C)** Changes in antibody titres on an hourly basis after the first dose of vaccine in one previously infected HCW (#5). During the previous infection, this HCW had mild symptoms. The upper left figure shows a magnified view of the data from baseline to 96 hours (day 4). COI, cut-off index; h, hour; D, day. The dotted line indicates the cut-off value (0.8 U/mL for anti-S and 1.0 COI for anti-N antibodies).

We next examined the kinetics of anti-S protein titres in four previously infected HCWs at baseline and 1, 2, 3, 4, 5, 6, and 7 weeks after the first dose of BNT162b2 mRNA vaccine (**Figures 1A** and **4A, B**; note that individual #1 had moderate COVID-19 symptoms and individuals #2, 3, and 4 had mild symptoms). All previously infected HCWs were seropositive for anti-S titres at baseline (median: 142 U/mL, range: 38–1941 U/mL) (**Figure 4**). The marked effect of boosting was observed as early as 1 week after the first dose in all previously infected HCWs (**Figure 4A**). The titres of anti-S protein antibodies increased 57.9-fold on median (range: 11.6- to 168-fold) at 1 week compared with baseline. Although the titres of anti-S protein antibodies subsequently showed a slight decreasing trend from 1 week to 4 weeks, an additional boosting effect was observed after the second dose (**Figure 4A**). The anti-S protein antibody titres increased a median of 2.5-fold (range: 1.7- to 4.4-fold) at 4 weeks compared with 3 weeks. The one previously infected HCW (individual #1) who had moderate COVID-19 symptoms during his prior infection had a dramatically higher increase in antibody titres following vaccination (1914 U/mL to 49500 U/mL) (**Figure 4A**).

Similar to the infection-naïve individuals, the anti-S antibody titres showed a decreasing trend after the second vaccine dose at weeks 6 and 7 (**Figure 4A**). Anti-N protein antibody titres were detectable over the entire study period and showed a slight decreasing trend (**Figure 4B**). These results indicated that a robust boosting effect was observed within 1 week after the first dose of the BNT162b2 mRNA vaccine in previously infected HCWs.

### Kinetics of Very Early Antibody Responses in a Previously Infected HCW

As mentioned above, a strong humoral response occurred within 1 week after the first vaccine dose in previously infected HCWs (**Figure 4A**). We further investigated the timing of increases in anti-S protein antibody titres using hourly blood sampling (6, 12, 24, 48, 72, 96, 120, 144, and 168 hours after the first dose) in one previously infected HCW (individual #5) (**Figure 1B**). Compared with baseline, anti-S protein antibody titres were unchanged from 6 h to 72 h (range: 223 U/mL to 264 U/mL) (**Figure 4C**). However, antibody titres started to increase at 96 h (Day 4) after the first dose and drastically increased thereafter (**Figure 4C**). This result indicated that activation of memory B cells to produce antibody occurred very early (e.g., within hours after vaccination) in previously infected HCWs.

## Discussion

A pervious study showed that COVID-19 mRNA vaccine causes antibody responses in infection-naïve and previously infected individuals (19–23), however, there are few studies elucidated the timing of the rapid immune response at specific time points, stratified by age and gender. In this study, we analysed serial blood samples from HCWs on a weekly basis before and after the BNT162b2 mRNA vaccination. The most striking finding was that a boosting effect of vaccination occurred in all individuals regardless of age or gender. In infection-naïve HCWs, we found that a further increase in antibody titre was observed one week after the second vaccination compared to immediately before the second vaccination. Hourly sampling revealed that the boosting effect could start as early as 96 hours (day 4) after the first dose of vaccine in previously infected individuals. Thus, the BNT162b2 mRNA vaccine is expected to provide a high level of protection against infection through the acquisition of immune memory and the extremely rapid boosting effect induced upon infection. This vaccine has been previously demonstrated to cause activation of memory B cells (18, 31). It is tempting to speculate from the results of this small study that the observed robust and rapid antibody response may be sufficient to protect against infection as well as to confer therapeutic effects. Emergence of high-titre antibodies within 96 hours may help to inhibit viral replication in the alveolar epithelium and minimise COVID-19 symptoms.

Interestingly, the kinetics of the boosting effect in infection-naïve and previously infected HCWs showed similar patterns. Among infection-naïve HCWs, humoral immunity was acquired after the first vaccination but at minimal levels, and a boosting effect was observed during the first week after the second dose. The important finding was that the boosting effect was similar in elderly and young individuals (**Figure 2B**). This boosting effect is expected to occur during natural infection as well in individuals who have received COVID-19 vaccinations. In previously infected HCWs, the boosting effect was observed during the first week after the first vaccine dose because these individuals had pre-existing immunological memory against the S protein. In previously infected HCWs, antibody titres increased only slightly after the second vaccination (**Figure 4A**), indicating that only one dose of vaccination was likely to be enough to induce a robust antibody response. Therefore, in the context of limited access to COVID-19 vaccines, a single dose of vaccine administered to previously infected individuals can be expected to save vaccine (20).

The present study presents the possibility that age and gender may influence differences in antibody response. In particular, the overall and stratified analyses showed that the age would affect the level of antibody titre. With regard to gender, the overall analysis showed a higher tendency in females, but the stratified analysis did not make a clear difference. A previous report shows that antibody titres are lower in elderly people (32). If this is true, it suggests that the decrease in antibody titres may be more noticeable in the elderly when time passes after vaccination, which may lead to a situation where additional vaccination should be considered.

Another major finding of this study was that a declining trend in antibody titres was already observed during the relatively short study period. Thus, it is conceivable that the antibody titres may decline to low levels within months or years. Therefore, there are concerns regarding the durability of protective humoral antibody to prevent future infections. To clarify this issue, larger numbers of individuals receiving the BNT162b2 mRNA vaccine and other types of SARS-CoV-2 vaccines would need to be followed prospectively over a longer period.

This study has some limitations, firstly the number of previously infected HCW is small (n=5) and all were young in their 20s and 30s. We observed a rapid antibody response within a week after the first vaccination in previously infected HCWs. For one HCW, antibody titre started to increase at 4 days by the analysis on an hourly basis. However, we consider that further data is necessary to make a more robust conclusion that antibody response occur within a week in previously infected individuals. Second, we didn’t measure neutralizing antibody responses, but rather measured total S and N antibody titres using the Elecsys Anti-SARS-CoV-2 assay (Roche Diagnostics). While anti-S antibody titre determined by this assay is associated with the antibody neutralizing ability (33), the level of neutralizing antibody needs to be examined for more accurate assessment. Third, more data is needed on the differences in the antibody titres by age and by gender. We observed that there was a significant difference between the higher antibody titres in infection naïve HCWs in their twenties than in their sixties, and in women than in men, when the whole group was analysed together (**Figures 3A, B**). However, stratified analysis of the respective data adjusting for gender and age groups did not yield statistically significant differences. The possible reason is that the sample size was small and the stratified analysis with adjustment did not provide statistical power for make a significant difference. Therefore, our finding should be considered preliminary data and will need to be validated by examining further cases in the future.

In summary, we demonstrated that early antibody responses and a marked boosting effect occurred in HCWs receiving the BNT162b2 mRNA vaccine. Our data showed a rapid development of the antibody response in a previously infected HCW as early as 96 hours (4 days) after the first dose. Similar short-term boosting effects may occur during natural infection and effectively suppress replication of the virus, blunting the infection and reducing the risk of severe symptoms.

## Data Availability Statement

The original contributions presented in the study are included in the article/**Supplementary Material**. Further inquiries can be directed to the corresponding author.

## Ethics Statement

The studies involving human participants were reviewed and approved by The Institutional Review Board of the Clinical Research and Genome Research Committee of Yamanashi Central Hospital. The patients/participants provided their written informed consent to participate in this study.

## Author Contributions

YH: Data curation, formal analysis, funding acquisition, investigation, visualisation, writing – original draft preparation, and writing – review and editing. KA: Data curation, formal analysis, and investigation. HS: Data curation and investigation. MS and MT: Project administration and resources. HM: Project administration and supervision. MO: Conceptualisation, supervision, and writing – review and editing. All authors contributed to the article and approved the submitted version.

## Funding

This study was supported by a Grant-in-Aid for the Genome Research Project from Yamanashi Prefecture (to YH and MO), a Grant-in-Aid for Early Career Scientists (18K16292 to YH), a Grant-in-Aid for Scientific Research (B) from the Japan Society for the Promotion of Science (JSPS) KAKENHI (20H03668 to YH), a Research Grant for Young Scholars funded by Yamanashi Prefecture (YH), the YASUDA Medical Foundation (to YH), the Uehara Memorial Foundation (YH), and Medical Research Grants from the Takeda Science Foundation (to YH).

## Conflict of Interest

The authors declare that the research was conducted in the absence of any commercial or financial relationships that could be construed as a potential conflict of interest.

## Publisher’s Note

All claims expressed in this article are solely those of the authors and do not necessarily represent those of their affiliated organizations, or those of the publisher, the editors and the reviewers. Any product that may be evaluated in this article, or claim that may be made by its manufacturer, is not guaranteed or endorsed by the publisher.

## Supplementary Material

Supplementary Figure 1 Priming effect of BNT162b2 mRNA vaccine in the infection-naïve individuals. The ratios of antibody titres before vaccination (baseline) and 3 week after the first dose (3 weeks) are indicated. **(A, B)** Data are shown for **(A)** age group and **(B)** gender. A pairwise t-test with Bonferroni correction was conducted for the multiple comparison test across the age groups. Student’s t-test was performed for statistical analyses between female (F) and male (M).

Supplementary Figure 2 Changes in anti-nucleocapsid (N) antibody titres in infection-naïve healthcare workers (n=103). Changes in antibody titres after the first vaccine dose from week 1 to week 7 in 103 healthcare workers (HCWs). The HCWs were seronegative throughout the observation period. COI, cut-off index. The dotted line indicates the cut-off value (1.0 COI).

Supplementary Figure 3 Stratified analysis by age and gender. **(A, B)** Stratified analysis of males and females in each age group. Data for females are shown in **(A)**, and data for males are shown in **(B)**. **(C–G)** Stratified analysis by age in gender. Data are shown for 1 week **(C)**, 3 weeks **(D)**, 4 weeks **(E)**, 5 weeks **(F)**, and 6 weeks **(G)**. Student’s t-test was performed for statistical analyses between female (F) and male (M). A pairwise t-test with Bonferroni correction was conducted for the multiple comparison test across the age groups. The dotted line indicates the cut-off value (0.8 U/mL).
