# Supplementary figures and images for: Robust Antibody Responses to the BNT162b2 mRNA Vaccine Occur Within a Week After the First Dose in Previously Infected Individuals and After the Second Dose in Uninfected Individuals
Source: Front Immunol. 2021 Aug 26;12:722766. doi: 10.3389/fimmu.2021.722766 (PMC8427169; doi:10.3389/fimmu.2021.722766)

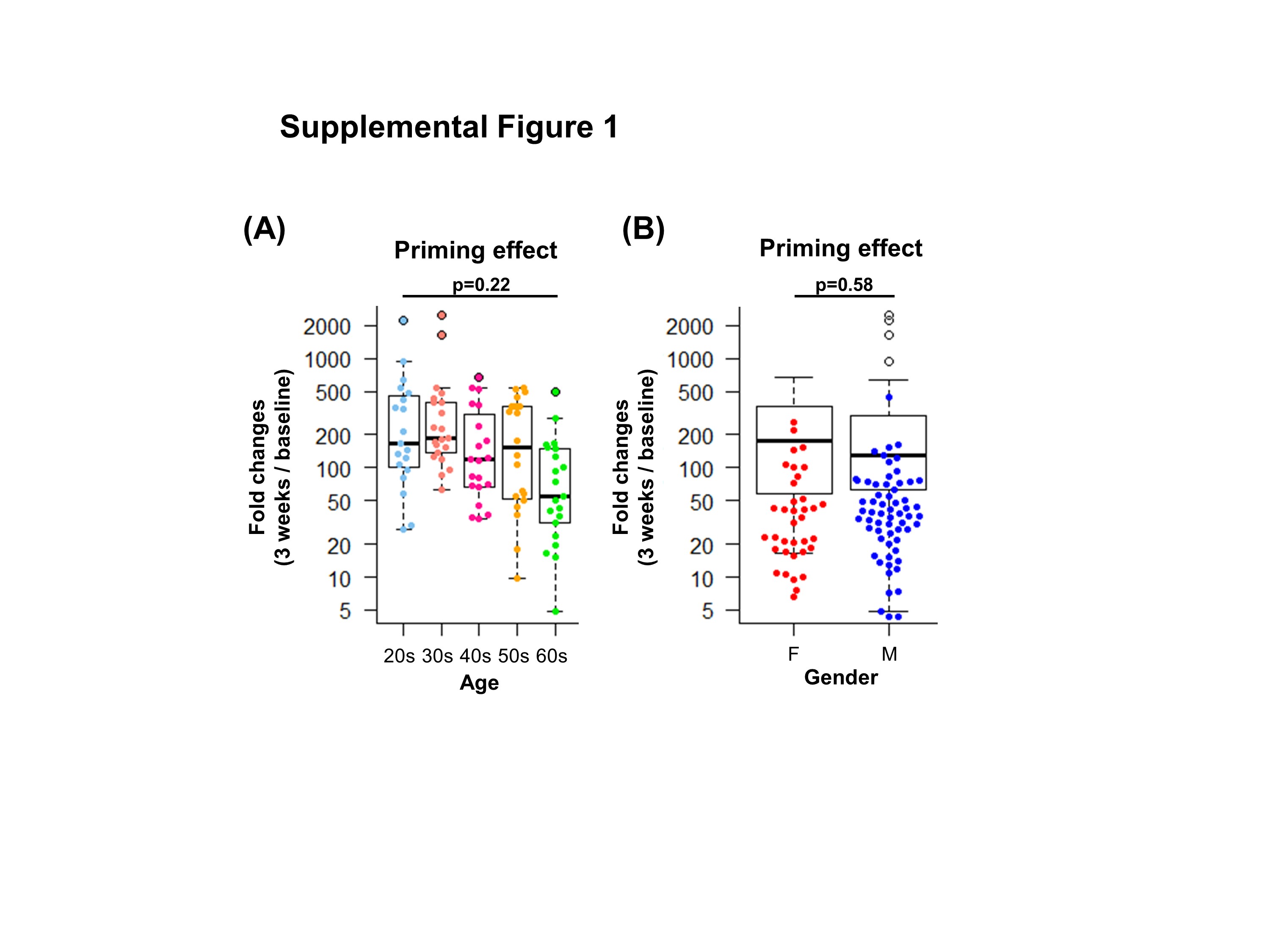

Supplement: Supplementary Figure 1 — Priming effect of BNT162b2 mRNA vaccine in the infection-naïve individuals. The ratios of antibody titres before vaccination (baseline) and 3 week after the first dose (3 weeks) are indicated. (A, B) Data are shown for (A) age group and (B) gender. A pairwise t-test with Bonferroni correction was conducted for the multiple comparison test across the age groups. Student’s t-test was performed for statistical analyses between female (F) and male (M). [file Image_1.jpeg]

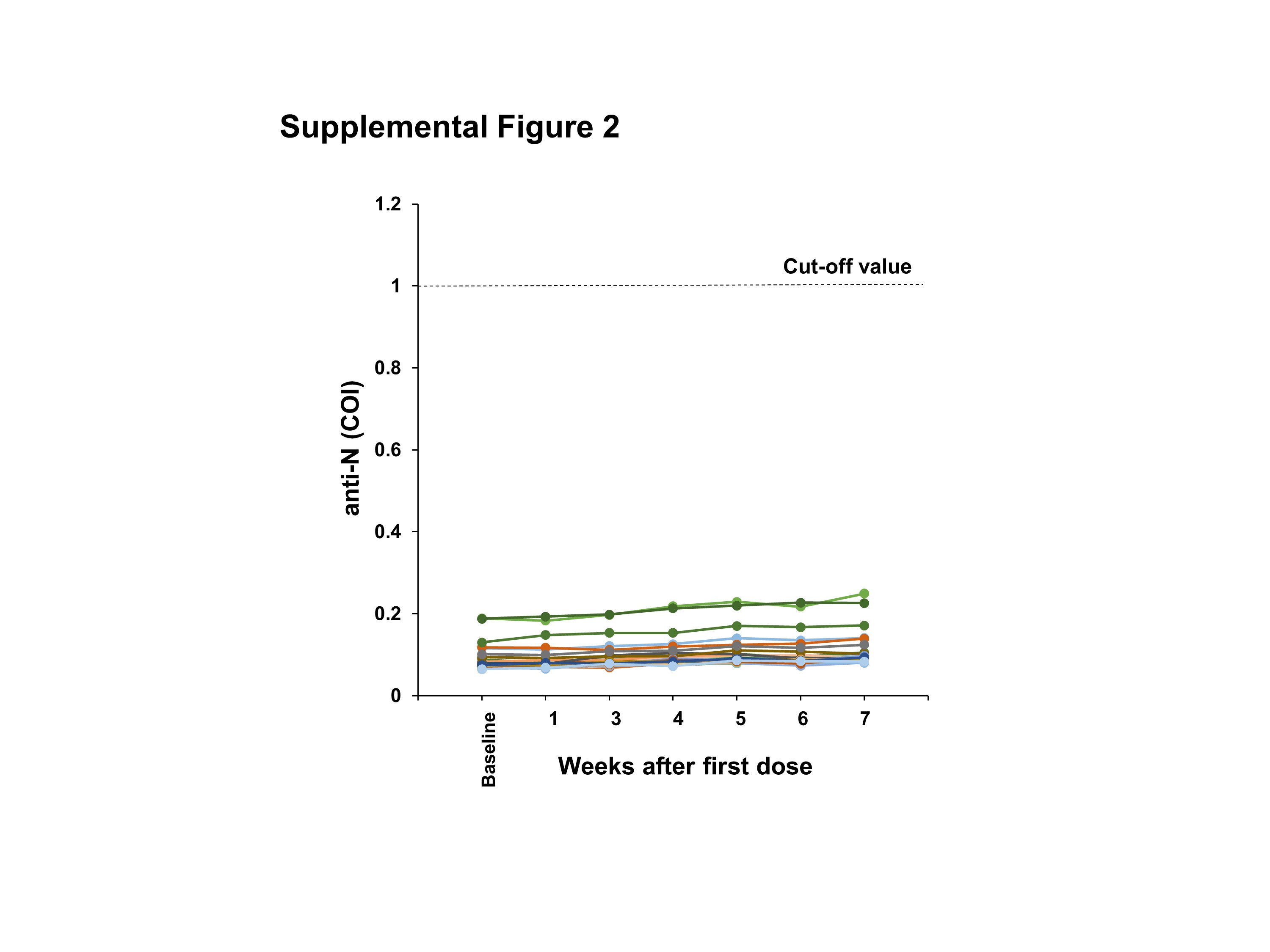

Supplement: Supplementary Figure 2 — Changes in anti-nucleocapsid (N) antibody titres in infection-naïve healthcare workers (n=103). Changes in antibody titres after the first vaccine dose from week 1 to week 7 in 103 healthcare workers (HCWs). The HCWs were seronegative throughout the observation period. COI, cut-off index. The dotted line indicates the cut-off value (1.0 COI). [file Image_2.jpeg]
