# Supplementary material for: Robust Antibody Responses to the BNT162b2 mRNA Vaccine Occur Within a Week After the First Dose in Previously Infected Individuals and After the Second Dose in Uninfected Individuals
Source: Front Immunol. 2021 Aug 26;12:722766. doi: 10.3389/fimmu.2021.722766 (PMC8427169; doi:10.3389/fimmu.2021.722766)

Supplemental Figure 3

The anti-spike antibody levels in female by age group

(A)

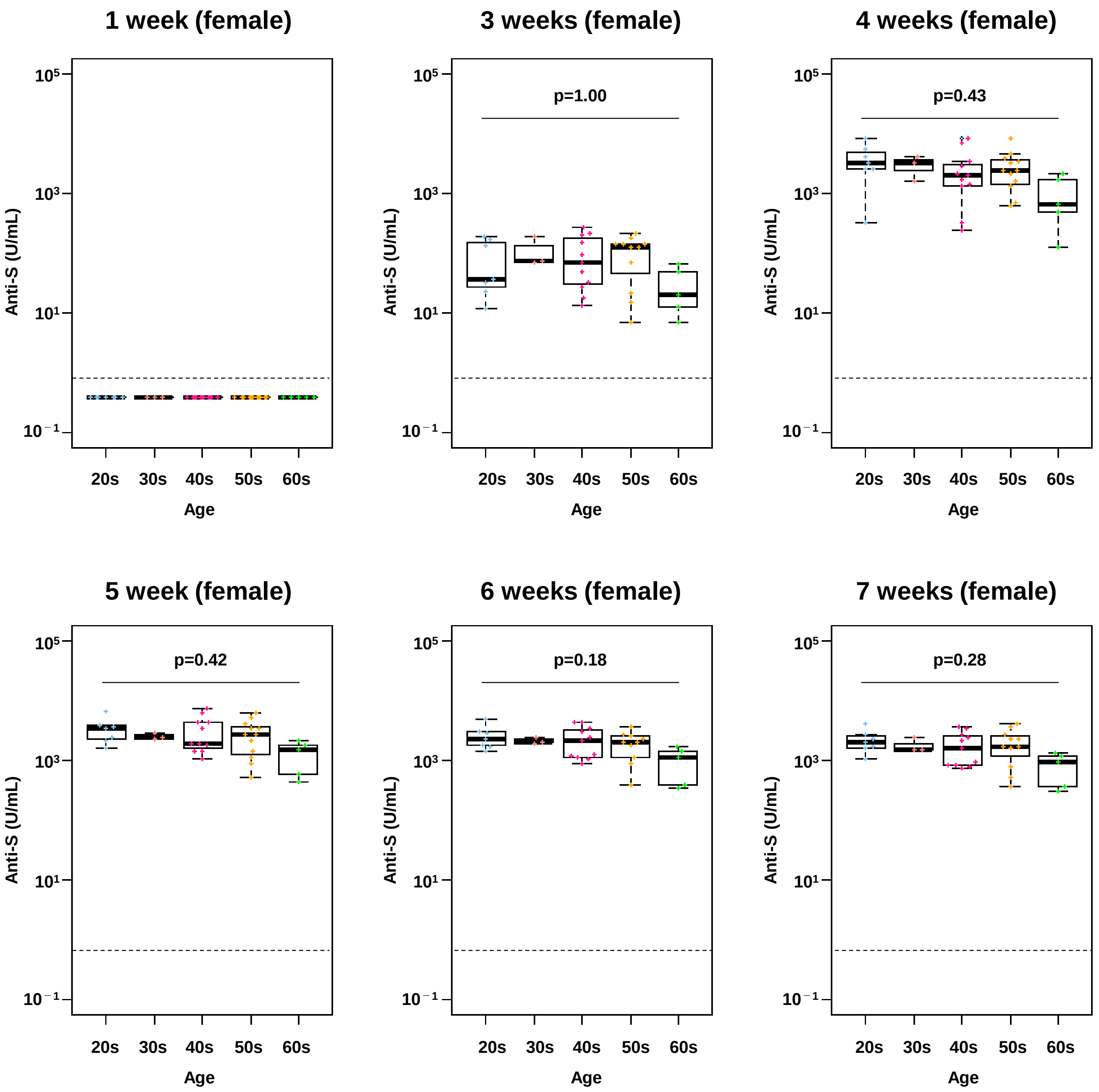

Supplemental Figure 3 (continued)

(B)

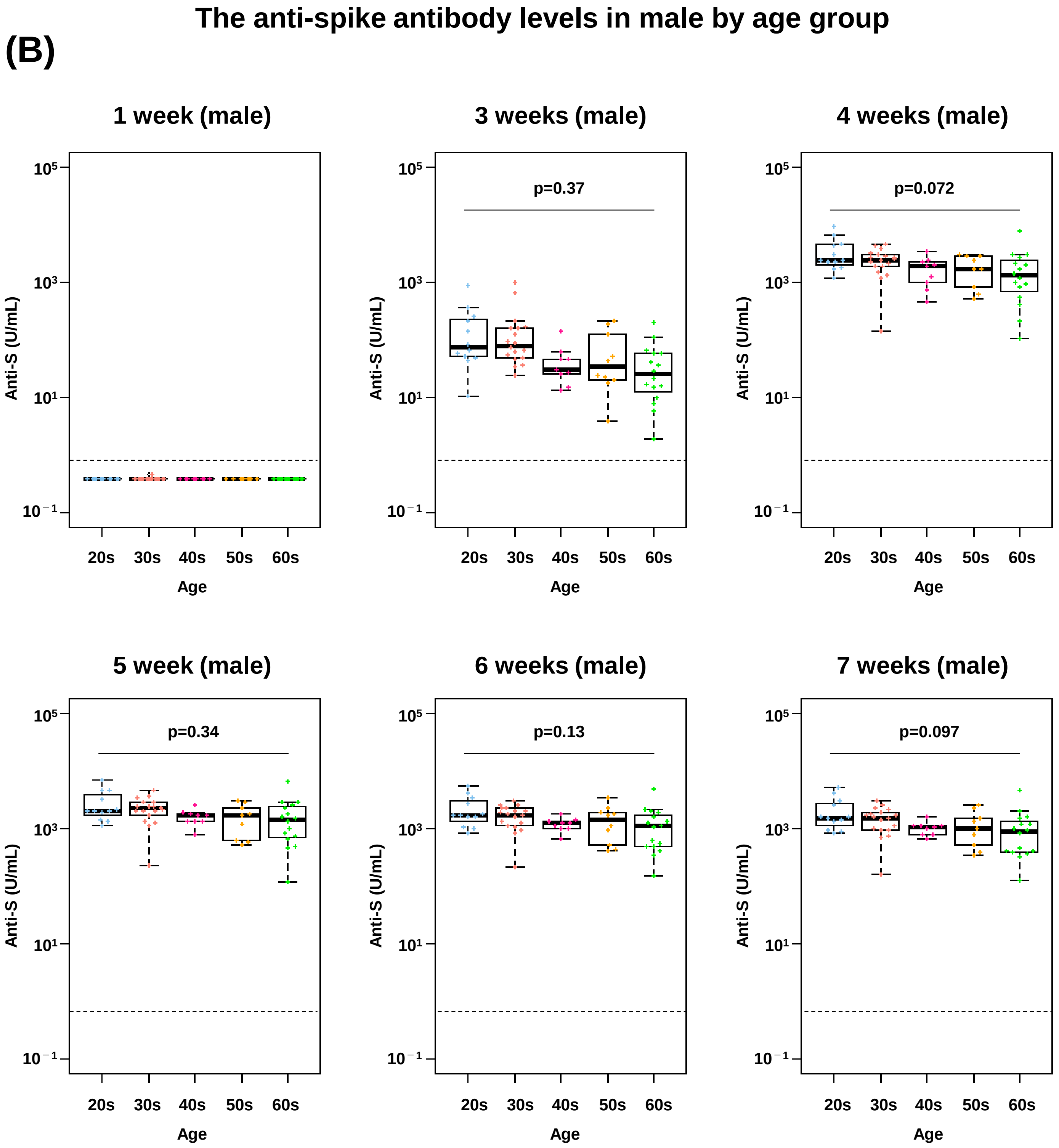

Supplemental Figure 3 (continued)

The anti-spike antibody levels in each age group by gender 1 week after first dose

(C)

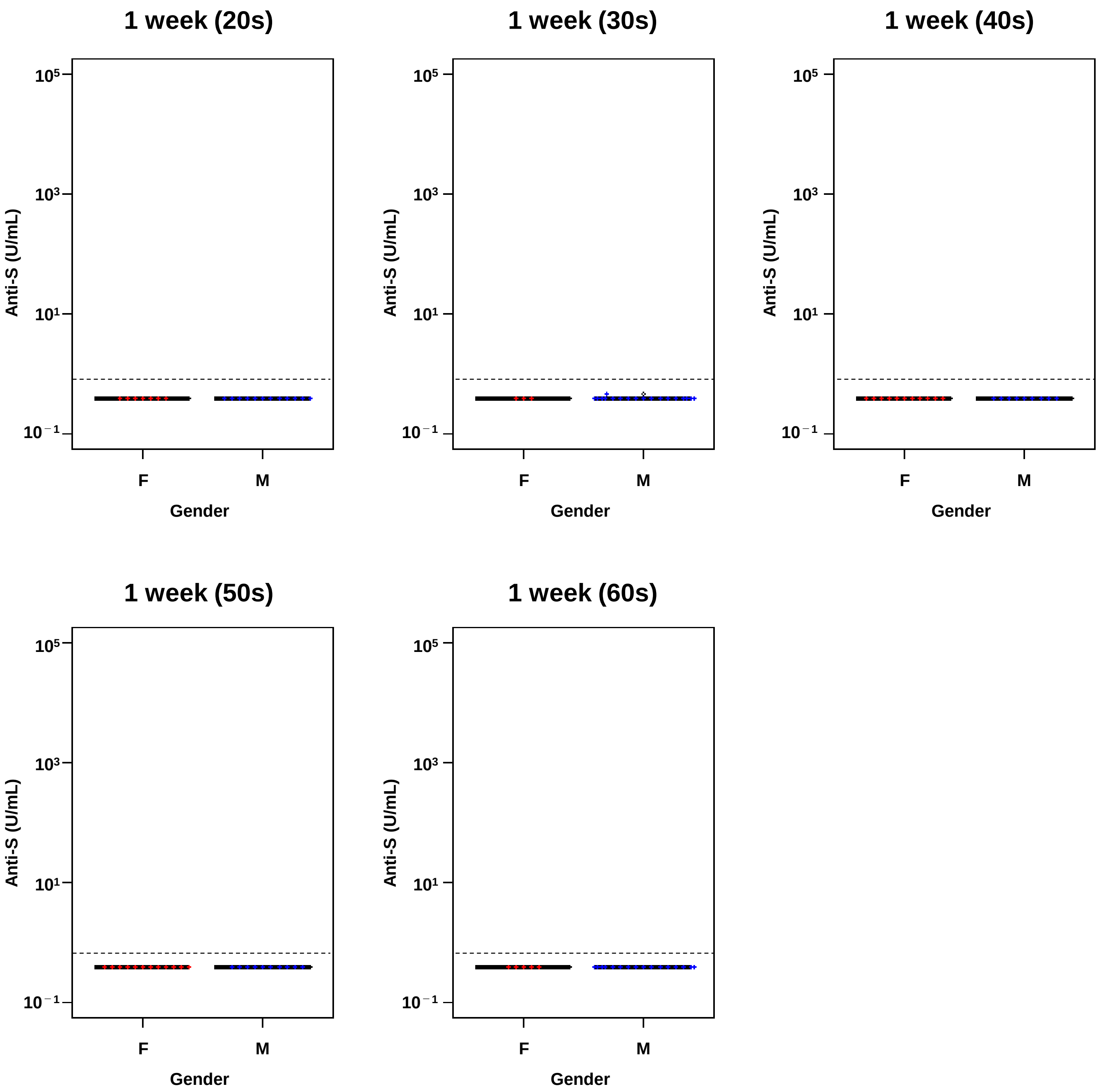

Supplemental Figure 3 (continued)

The anti-spike antibody levels in each age group by gender 3 weeks after first dose

(D)

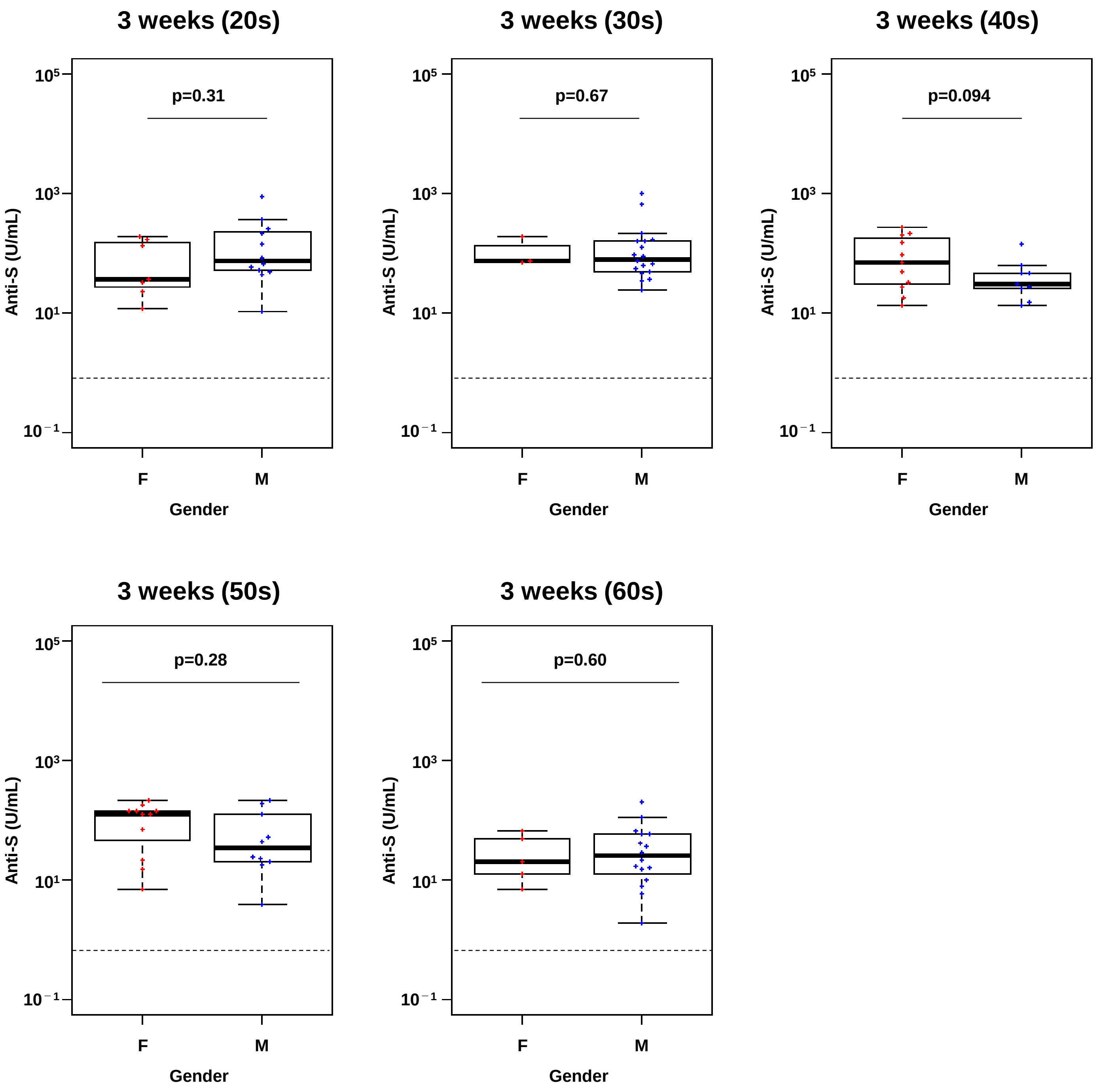

Supplemental Figure 3 (continued)

The anti-spike antibody levels in each age group by gender 4 weeks after first dose

(E)

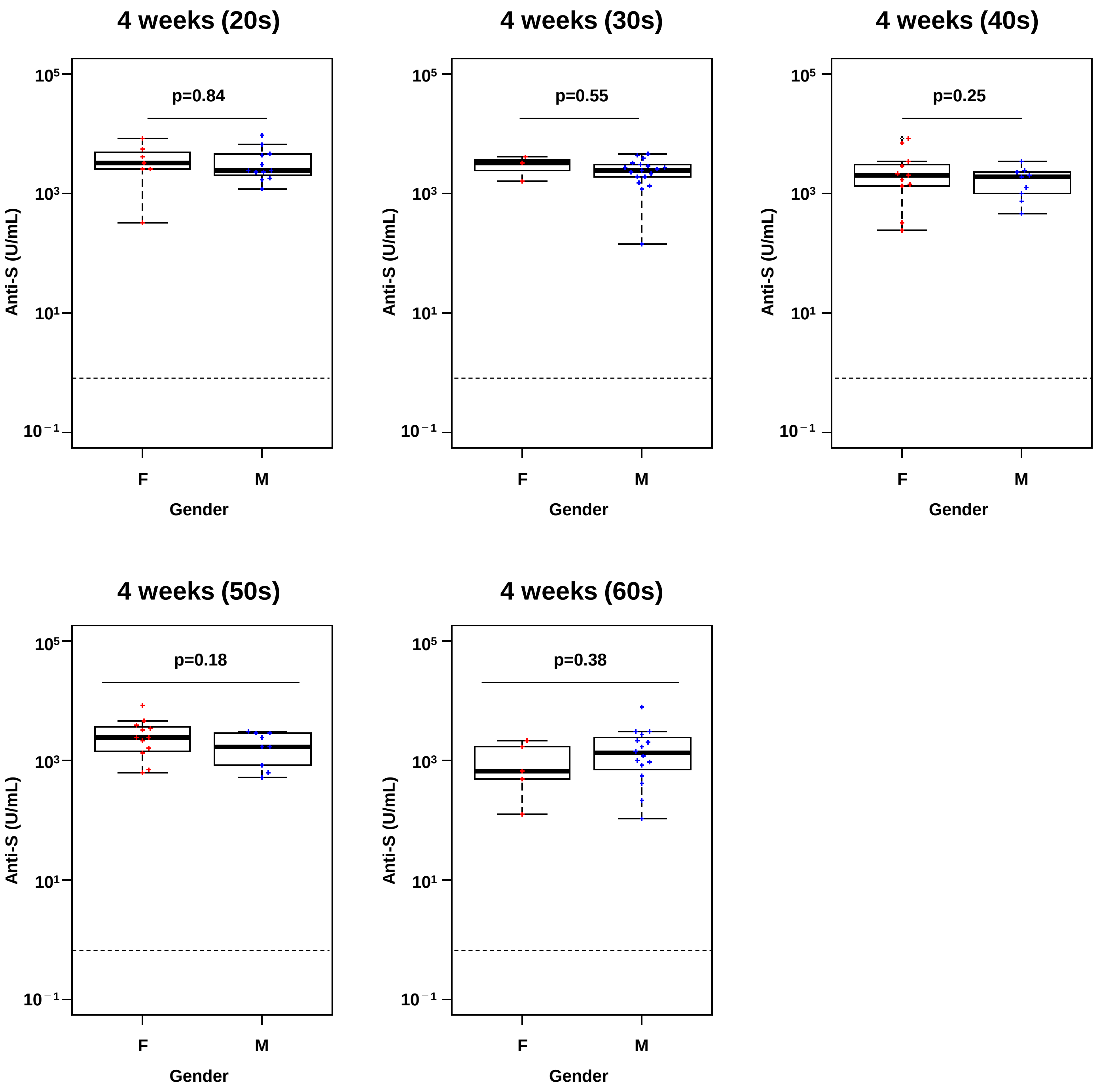

Supplemental Figure 3 (continued)

(F) The anti-spike antibody levels in each age group by gender 5 weeks after first dose

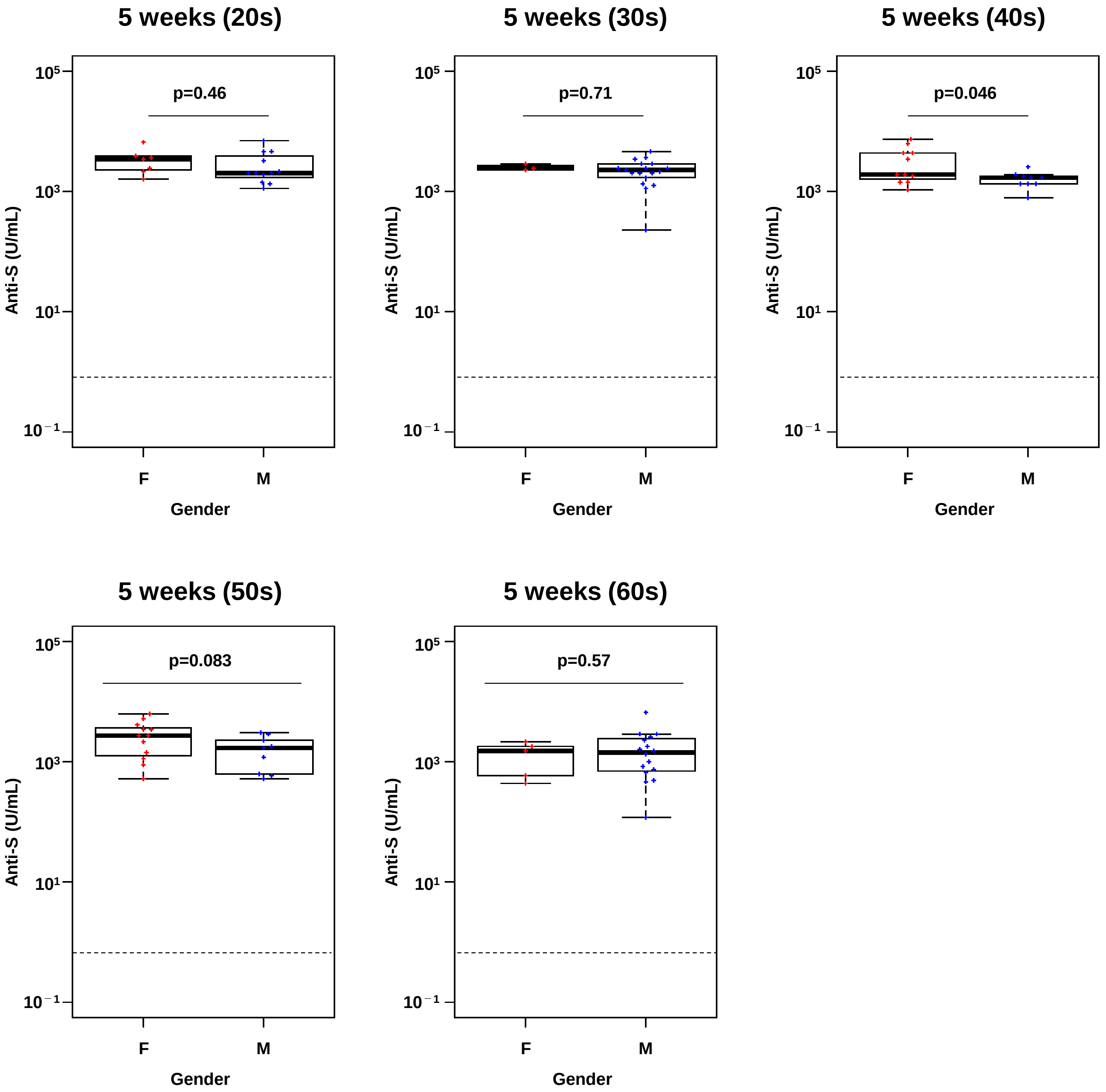

Supplemental Figure 3 (continued)

The anti-spike antibody levels in each age group by gender
6 weeks after first dose

(G)

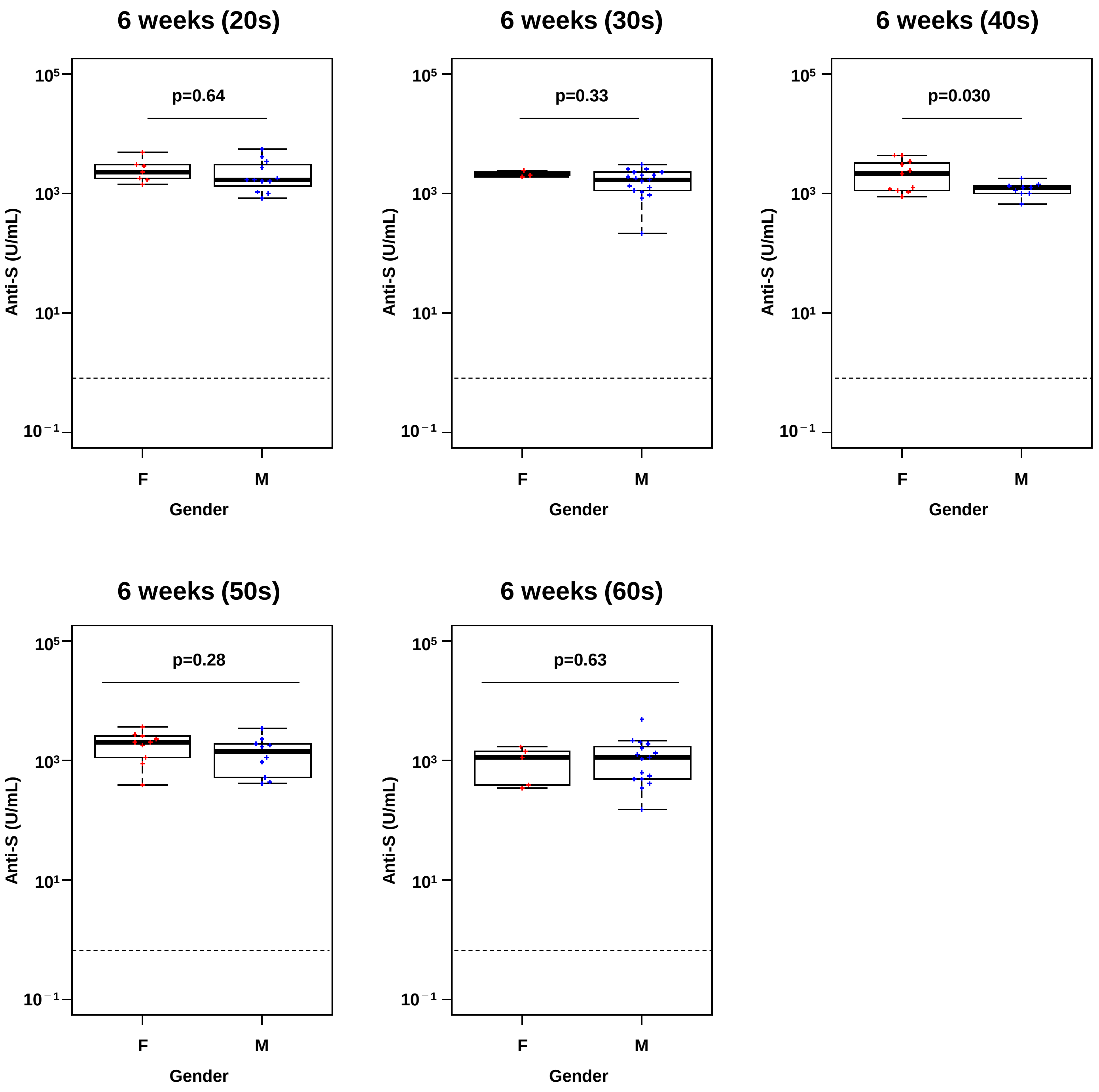

Supplement: Supplementary Figure 3 — Stratified analysis by age and gender. (A, B) Stratified analysis of males and females in each age group. Data for females are shown in (A), and data for males are shown in (B). (C–G) Stratified analysis by age in gender. Data are shown for 1 week (C), 3 weeks (D), 4 weeks (E), 5 weeks (F), and 6 weeks (G). Student’s t-test was performed for statistical analyses between female (F) and male (M). A pairwise t-test with Bonferroni correction was conducted for the multiple comparison test across the age groups. The dotted line indicates the cut-off value (0.8 U/mL). [file Image_3.pdf]
